# Training for Coherence Formation When Learning From Text and Picture and the Interplay With Learners’ Prior Knowledge

**DOI:** 10.3389/fpsyg.2019.00193

**Published:** 2019-02-07

**Authors:** Tina Seufert

**Affiliations:** Department for Learning and Instruction, Institute of Psychology and Education, Ulm University, Ulm, Germany

**Keywords:** coherence formation, multimedia learning, text-picture-integration, effects of prior knowledge, aptitude-treatment-interaction

## Abstract

Learning with text and pictures requires learners to integrate the given information into one coherent mental representation. Since learners often fail to integrate text and pictures, the study investigates the effects of a training for text processing strategies, picture processing strategies and strategies to map text and picture onto each other. It was assumed that learners’ prior knowledge would affect the effects of such a training with more beneficial effects for learners with high prior knowledge. The training comprised an introduction on how to process, integrate and reflect on texts and pictures with an additional training phase of 3 weeks. The study (*N* = 30) analyzed the effects of the training with regard to recall and comprehension performance in contrast to the no training group, which received an alternative program that was not related to text-picture integration. A regression analysis showed that the integration training was not overall beneficial but only for learners with increased levels of prior knowledge. Hence, training for coherence formation is beneficial for learning only when adequate knowledge structures are available to conduct the recommended steps of understanding and integrating text and picture.

## Introduction and Theoretical Background

When taking a look at modern learning material in books, on websites, in learning apps or conveyed by teachers, the most prominent presentation formats are texts accompanied by pictures. While texts are used to provide facts and details, pictures are often used to illustrate the topic and to provide an overview. In biology textbooks, for example, texts describe the processes that take place within a human cellular system like movements or transactions. The accompanying picture complements the understanding of these processes by providing an overview of the cellular structures. Together, text and picture help to understand a complex learning topic and both forms of representations convey different but interdependent information that has to be linked in the learner’s mind (Ainsworth, 2006).

There is large evidence on the so called multimedia effect that when learning from text and picture learners’ recall and comprehension performance is in fact higher than when learning from text alone (for an overview see Butcher, 2014). This fostering effect can be explained with Paivio’s Dual Coding Theory (Paivio, 1986), i.e., text and pictures are first processed in separate memory systems and hence lead to two separate memory traces. This dual coding of information enhances the probability to retrieve information from long-term memory. The positive effect on conceptual understanding or even on transfer of knowledge nevertheless depends on the above-mentioned integration of text and picture into one coherent representation within the learner’s mental system (Mayer, 2009; see also Seufert, 2003; Scheiter et al., 2017). Hence, it is worth taking a closer look at the cognitive processes of integrating text and picture and their specific challenges in detail.

### Learning From Text and Pictures

The most prominent model that describes processing of text and picture is Mayer’s Cognitive Theory of Multimedia Learning (CTML; Mayer, 2009). Based on Paivio’s dual coding assumption (1986), information from text and picture is first selected in two separate subsystems. The next process of organizing requires the association of information within the text and picture subsystem, and hence encompasses the construction of separate mental models of the two information sources. In a last step, the information of the two mental models is integrated into one coherent mental representation by using prior knowledge stored in long-term memory. Mayer (1997) specifies this process of integration as referential processing by one-to-one-mappings. Thus, corresponding elements and relations of the single representations are related to each other in order to extract the underlying structure of the two representations together.

The model of integrative text and picture comprehension (ITPC) from Schnotz and Bannert (2003) also states separate processing systems for textual and pictorial information. However, the model especially differentiates the affordances of text processing in different steps. Learners first have to syntactically process the information without necessarily understanding the meaning. Only in a second step a semantic analysis helps to extract the meaning and leads to the deduction of propositions. These are then connected in a propositional network representation, which is still verbally coded. In the last step learners construct an analog mental model. Symbolic information therefore has to be translated into analog information. The external picture is also an analog representation and therefore using the picture as a scaffold can ease the construction of the mental model. Based on ITPC, the integration of text and picture information into one mental model means that the analog structure of the picture can build the frame for the mental model, which is then enriched by propositions from the text and is connected to learners’ prior knowledge. Overall, in both models the processes of coherence formation and integration are accompanied by top down processes, i.e., by using prior knowledge.

### Integrating Text and Picture

Both models describe the process of learning from text and picture and highlight the necessity to mentally integrate both sources. However, there is still no explicit model that describes the process of integration. Based on Mayer’s (1997) description of one-to-one-mapping, the integration process can be seen as a process of structure mapping. Learners identify relevant concepts or statements in the text and picture, compare them and link them if possible. The idea of identifying and linking corresponding elements is also explained in models of understanding multiple documents (for an overview see Barzilai et al., 2018). With reference to Gentner’s (1983) structure mapping theory, Seufert and Brünken (2006) describe the process of integrating text and pictures, or multiple representations in general, as a process of mapping elements or relations in order to construct a coherent mental representation. Thus, this process is called coherence formation. Learners have to find corresponding elements that can be mapped onto each other (element-to-element mapping). Moreover, more comprehensive structures of elements and their interrelations have to be mapped onto each other (relation-to-relation-mapping). With reference to ITPC, one essential aspect for connecting representational structures is to translate between different sign systems. For example on the one hand, a pictorial element has to be verbalized in order to be able to relate it to other verbal elements. And on the other hand, verbal items must be translated into graphical structures in order to integrate them into one overall mental picture (e.g., Schnotz and Bannert, 2003).

Moreover, according to Seufert (2003) the mapping and translation processes can be conducted on different levels; syntactically or semantically. Information can only be processed superficially in order to extract the relevant surface features, e.g., shape or color (in a picture) or nouns and verbs (in a text). Hence, when a learner identifies corresponding surface features (e.g., when important parts are marked in red in the picture as well as in the text) and uses them as a hint for integration, this kind of mapping is called *syntactic mapping*. However, this does not necessarily come along with a deeper understanding of the single representations and consequently it does not ensure a comprehension of the overall relations. Instead, it would be desirable to animate learners to *semantic mapping*: In this case, elements and relations of single representations are mapped onto each other because learners really understand the semantic correspondences. Consequently, the resulting integrated knowledge structure is coherent and a basis for deeper understanding, appliance and transfer processes (Seufert, 2003).

It is obvious that finding relevant elements and relations within text and picture in order to map them onto each other can be a complex and effortful process for learners. This is especially the case when learners are not even able to identify what is relevant within the text or picture due to a lack of prior knowledge or strategies to understand texts or pictures. Thus, integrating text and picture on a semantic level has the potential to cause difficulties. There is evidence from eye-tracking studies that learners could in fact gain better learning outcomes when they show intensive transitions between text and pictures (Hegarty and Just, 1993; Scheiter and Eitel, 2015; Schüler, 2017) but that only a part of the learners actually showed such integrative behavior (Mason et al., 2013). Very often, learners pay attention to texts while they only briefly regard the pictures (Hannus and Hyönä, 1999). Hence, they fail to successfully integrate both sources. Renkl and Scheiter (2017) also underline the challenges of integrating visual displays with other representations.

Consequently, there have been a lot of studies during the last decade dealing with the possibilities to foster learning with text and picture in order to cope with the difficulties and to profit from potential positive effects of an integrated mental representation.

### Fostering Text-Picture-Integration

In general, there are two possible strategies to foster text-picture-integration. The first one is to add additional information like signals or explanations that help learners to identify corresponding elements in text and picture. Corresponding colors, connecting lines or the spatial integration of texts within pictures have frequently been used as signals for correspondences. A meta-analysis of Richter et al. (2016) on signaling revealed a small to medium fostering effect of signals on learning performance. Nevertheless, signals can only give hints of what could be mapped onto each other. Therefore, they are only low-key prompts for integration on a surface level. To ensure that learners actually engage in structure mapping on a semantic level, one could provide explicit information about the correspondences between representations (e.g., Seufert and Brünken, 2006). Such explicit explanations of semantic references turned out to be helpful, especially when combined with signals on the surface feature level (Seufert and Brünken, 2006). Instead of providing explanations on references, learners could also be prompted to find correspondences themselves. Studies in which integration prompts were used revealed positive effects on learning (e.g., Bodemer et al., 2005; Leopold et al., 2015). There is nevertheless evidence that learners can only profit from prompts, if the references they draw are actually correct (Leopold et al., 2015). Bodemer and Faust (2006) also could confirm that a drag and drop-integration task did not foster learning due to erroneous connections made. This was especially the case for learners with low levels of prior knowledge. These results reveal the shortcomings of prompts as a mean to foster text-picture integration. They can only help to overcome a production deficiency, i.e., learners are prompted to conduct a procedure they already know and master (Bannert, 2009). The reported studies nevertheless indicate that learners are not necessarily able to successfully integrate.

This leads to the second principal approach to foster the integration of text and picture: training learners to implement a successful strategy of coherence formation. With such a training one could enable learners to deal with text and pictures in general. Hence, the training would be more enduring and easier to transfer (e.g., Dignath et al., 2008).

### Training to Integrate Text and Picture

A training that helps learners to integrate text and pictures can be seen as a training of a strategy that can be used for every combination of text and picture. Based on a vast amount of training studies for learning strategies in general (for an overview see Dignath et al., 2008), one can determine crucial issues for effective strategy trainings. The first crucial issue is based on studies on training of cognitive strategies which point out that single elements of the strategy have to be mastered before the separate parts can be combined into one complex skill (McNamara et al., 2004). The second one is that learners should be provided with the crucial steps of the strategy, i.e., the cognitive aspects of the strategy as well as metacognitive strategies to regulate their strategy use (Berthold et al., 2007). When conducting the separate steps of a complex strategy, learners are then able to monitor their progress and can readjust their behavior, thus improving their strategy skills.

While these recommendations are crucial for strategy trainings in general, the above mentioned models of text-picture integration (Schnotz and Bannert, 2003; Mayer, 2009) as well as the concept of coherence formation as structure mapping (Seufert, 2003) provide specific guidelines for developing a training for text-picture integration.

As text and picture are processed separately in the first place, the trained strategy should comprise specific steps for text processing and picture processing. According to Mayer (2009), *text processing* starts with a selection process which, according to the ITPC model, mainly refers to surface features of the text. Thus, learners could start with getting an overview by scanning the headlines, the structure with its columns or chapters and first sentences. This bottom-up-process should be accompanied by top-down-processes. Thus, learners should activate their prior knowledge by reflecting on what they already know about the content. This fosters the process of organizing the information and deducing relevant propositions out of the text. Thereby, the crucial part of text processing strategies is to identify the relevant elements and relations that are the basis for subsequent mapping processes (e.g., McNamara et al., 2004). The last step, the construction of a mental model then requires learners to integrate the identified separate aspects into one coherent mental representation of the text, again by linking the text content to their prior knowledge. With reference to the ITPC model this last step of a mental model construction requires learners to mentally translate the verbal content into an analog mental structure. This process can be eased particularly when learners are prompted to self-explain the overall meaning of the text in their own words (McNamara et al., 2006).

The same strategy could be used for *picture processing*. Learners have to scan the picture to grasp the overall spatial structure. There is evidence that a first glance at the picture – and even a very short one – turned out to be highly effective for later text-picture processing (Eitel et al., 2013). The authors argue that learners can use the external picture as a scaffold for mental model construction. Learners then again should activate their prior knowledge in order to foster the selection and organization process. Due to their analog nature, pictures are often only viewed as one unit in a superficial way. Hence, Stalbovs et al. (2015) stress the importance of decomposing pictures into meaningful parts. This step should therefore be assisted by the instruction to identify and mark relevant elements and relations. However, for organizing and constructing a mental model of the picture content, learners need to bring the elements and relations together again into one meaningful unit and they have to draw inferences from the picture (Hegarty, 2005).

However, the picture processing strategy and especially the steps of identifying relevant elements and relations differ notably for either realistic or logical pictures (Schnotz and Bannert, 2003). Thus, learners have to be provided with additional meta-representational information about the features of realistic pictures and with reference to the study of Miller et al. (2016) especially of logical pictures. They found that providing information about the conventions of diagrams in short warming-up tasks could increase understanding of logical pictures.

When learners possess effective strategies to deeply process and understand the text as well as the picture, the prerequisites for *structure mapping*, and hence for *integration* are given. With reference to the differentiation into surface and semantically oriented mapping processes (Seufert and Brünken, 2006) the learners could again start to use the surface features they extracted for their mapping process, like headlines or salient features. CTML as well as the ITPC model point out that the integration process is facilitated by using prior knowledge. Thus, the mapping strategy should also start with the activation of prior knowledge with respect to the overall content. This also enables semantic mapping processes and the linking of corresponding elements as well as relations between text and picture. As mentioned above, meaningful mapping is the crucial step for successful learning with text and picture. This is underlined by the eye-tracking study of Mason et al. (2013) where learners who integrated text and picture by looking intensively back and forth outperformed low-integrators.

Given that a training for text-picture integration should equip learners to deal with every possible combination of text and pictures learners should also learn how to evaluate the representational functions of text and picture. Based on Ainsworth’s DEFT model (2006), representations can serve different functions. They can, for example, complement or constrain each other or they can even be redundant. Thus, as a part of the mapping strategy learners should evaluate whether text and picture or parts of them are redundant or complementary or whether there are parts that do not refer to the other representation or that are maybe even irrelevant. This reflection helps learners to gain meta-representational knowledge about texts and pictures and their functions. Schwonke et al. (2009) could show that providing such information about representational characteristics can deepen learners understanding and intensify the mapping process.

There is evidence from two previous studies that such a comprehensive training or support for integrating text and pictures can foster learning. Schlag and Ploetzner (2011) conveyed a short presentation of a step-by-step plan for text-picture-processing in their study and let learners practice these steps afterwards. The training turned out to be effective compared to a control group without training for all levels of understanding (factual, conceptual and transfer). The second study from Stalbovs et al. (2015) used implementation intentions as a specific strategy to support the essential steps of text-picture-integration. Learners should internalize if-then-plans, so that whenever they are in a specific situation (e.g., if I have opened a new page) they will conduct a specific operation (e.g., then I will carefully study the title first). Stalbovs et al. (2015) instructed learners to internalize different variations of such implementation intentions that either addressed deepened text-, picture- or text-and-picture-processing. Learners were best supported when all three aspects were covered by the implementation intentions, which will also be the case in our training.

Thus, overall there is evidence that learning from text and pictures can be improved when learners are provided with a strategy training that comprises the crucial steps of text-processing, picture-processing and text-picture integration. However, based on prior studies on the effectiveness of help for coherence formation when learning from multiple representations one can ask whether training effects might depend on learners’ prior knowledge.

### Aptitude-Treatment-Interaction Effect of Help for Coherence Formation

Considering the affordances of the above mentioned strategies to understand text and picture and to integrate them, learners should be able to identify and map relevant elements between text and picture. With insufficient prior knowledge in the domain, learners lack appropriate cognitive schemata to identify the relevant elements in the single representations. In scientific domains and mathematics, it has often been proven that learners only concentrate on surface features (for an overview see Ainsworth, 2006) and hence cannot map between the representations semantically. They also often face problems with translating between different representational codes (Baker et al., 2001). It is also plausible that novice learners experience increased intrinsic cognitive load because they cannot build meaningful chunks (Bannert, 2002). Thus, their cognitive resources can easily be overloaded when they try to meaningfully integrate text and pictures. To additionally handle an unknown strategy could be even more strenuous and thus a strategy training might not be effective (Bjorklund and Coyle, 1995). These theoretical assumptions are in line with empirical results on the effectiveness of help for coherence formation. Based on a study of Seufert (2003), one can assume that prior knowledge is actually relevant for the effectiveness of help for coherence formation. The study revealed that the hints for integrating different representations only turned out to be effective for learners with a medium level of prior knowledge, whereas learners with too low or too high levels of prior knowledge did not improve when help was provided. The paper argues that especially novices lack the abilities to use such help adequately even though they would need it (see also Bodemer and Faust, 2006). Learners with high levels of prior knowledge also do not profit from help because they do not need it any longer. Only learners with a medium level of prior knowledge will still need some assistance and have enough resources and conceptual background to use the help effectively. They will be met in their zone of proximal development (Vygotsky, 1978) where help can effectively be used to accomplish the next level of expertise. In the present study it will be analyzed whether these moderating effects can also be revealed when learners are provided with coherence formation strategies in a pre-training.

## Research Questions and Hypotheses

The present study investigates, whether strategies for integrating text and picture can effectively be conveyed in a pre-training, and whether the effects of training depend on learners’ prior domain specific knowledge.

Based on the different levels of processing when dealing with text and picture, the training was designed to be helpful for both, recall and comprehension. But as the strategy especially aims at integrating text and picture on a semantic level, the effects should be stronger on comprehension as a higher level of processing.

However, the training is not assumed to be effective in general. Instead, learner’s prior knowledge should affect the effectiveness of the training. Only with a sufficient level of prior knowledge should learners be able to apply the strategy. Their existing schemata will help to identify the relevant elements and relations in text and picture and to map them onto each other on a semantic level. Learners with lower levels of prior knowledge should have difficulties in applying the strategy and even if they manage to extract and map relevant information, they might not be able to build semantically meaningful chunks. To handle the newly acquired strategy would pose additional load. Consequently, the training might even be harmful for them compared to a no-training condition. In terms of Mayer (1997), we hypothesize an enhancing effect of prior knowledge for the effectiveness of the training. If the sample would also include experts with high levels of prior knowledge one could expect no or even detrimental effects for them, as they should be able to accomplish the integration task without any further help. The additional information about implementing the strategies could thus lead to an additional mental effort, as the experts would have to actively ignore it. Thus, the so-called expertise reversal effect (Kalyuga, 2007) could be expected.

## Materials and Methods

### Participants and Design

Thirty university students of psychology and teacher training programs participated in the experiment. 14 of them were female and the average age was 23.53 (*SD* = 3.25). The sample only comprised learners with low to medium prior knowledge. Thus, no expertise-reversal effect will be analyzed as there were no expert learners in the given sample.

Participants were randomly assigned to one of the two treatment groups [experimental group with training (EG; *n* = 15) and control group without training (CG; *n* = 15)]. As dependent variables learners’ performance was measured, differentiated for recall and comprehension.

In a linear regression analysis the effects of the treatment as a categorical factor (with or without training), prior knowledge as a continuous factor, and the interaction of both by including the product of both variables were analyzed. Learners’ spatial abilities and working memory capacity were correlated with the performance measures and thus were included in the model as covariates.

### Materials and Procedure

The experiment was part of an advanced seminar in educational science. Students, nevertheless, could decide for themselves whether they wanted to participate in the study or not. The experiment was conducted in three sessions: one pretest session, the training session (or the alternative session for the control group) with a 3 weeks practicing phase afterwards and the posttest in a separate session to prevent exhaustion.

#### Pretest Session

In the pretest session, we analyzed prior knowledge of the content domain, which will be used in the posttest (the function of an Otto engine) with 4 open questions and one picture-labeling task. The prior knowledge test comprised questions on a recall level, like “Name the 4 strokes of the Otto engine cycle” and on a comprehension level, like “explain the two processes that causes the warming of the air-fuel-mixture.” Maximally 11.5 points could be reached and Cronbach’s α = 0.84 was sufficient. Additionally, a test for spatial abilities (% correct) was conducted (Paper Folding and Card Rotation test; Ekstrom et al., 1976). At last, working memory capacity was assessed (memory updating numerical, Oberauer et al., 2000). The score in this test reflects the number of related elements learners can process simultaneously and it usually ranges from 1 to 6 (with a theoretical maximum of nine). Overall, all pretests took about 1 h.

#### Training Program for the Experimental Group

The training consisted of a training session and a 3 weeks practice phase afterwards. The training session took place 1 week after the pretest and lasted 90 min. The students of the experimental group worked individually with a workbook to train the coherence formation strategy. The individual learning phase allowed individual pacing. The workbook comprised three strategy parts: (1) a text reading strategy, (2) a picture reading strategy for realistic and logical pictures and (3) a strategy for integrating texts and pictures. For each of these strategies the workbook provided a step-by step explanation of how to apply the strategy. The different steps are outlined in Table 1.

**Table 1:** Overview of the trained strategies and their crucial steps.

Type of strategy	Strategy steps	Rationale for strategy steps
Text reading	Get a first overview	Scanning/bottom-up
	Think of what you already know	Activating prior knowledge/top-down
	Read the text and mark relevant words	Identifying relevant elements
	Re-read the text, mark relevant sections and annotate them with short summaries	Identifying relevant relations
	Summarize the main statements of the text with reference to your prior knowledge either verbally or by sketching a picture	Structuring and elaborating to ensure understanding and long term recall
Picture reading	Identify the type of picture	Sensitize for different types of pictures
	Get a first overview	Scanning/bottom-up
	Think of what you already know	Activating prior knowledge/top-down
	Search the picture for relevant picture parts (e.g., figure-ground and highlighting in realistic pictures/a legend, labels of axis, salient features in logical pictures)	Identifying relevant elements
	Look for overall structures in the picture (e.g., interlinked picture parts in realistic pictures/slopes or contrasts of trends for logical pictures)	Identifying relevant relations
	Summarize the main statement of the picture with reference to your prior knowledge and write it down	Structuring and elaborating to ensure understanding and long term recall
Text-picture integration	Get a first overview by reading the title of the text and scanning the picture	Scanning/bottom-up
	Think of what you already know	Activating prior knowledge/top-down
	Read and understand the text by using the text reading strategy	Text processing
	Examine and understand the picture by using the picture reading strategy	Picture processing
	Identify corresponding elements in text and picture	Element- to-element-mapping
	Identify corresponding statements/structures in text and picture	Relation-to-relation-mapping
	Identify complementary elements or statements/structures in text and picture	Evaluating the function/relation of text and picture
	Summarize the main statement of the text-picture-combination with reference to your prior knowledge and write it down	Structuring and elaborating to ensure understanding and long term recall
Overall	Using the strategies checklist as a guideline and reflection tool	Metacognitive monitoring

**FIGURE 1:**
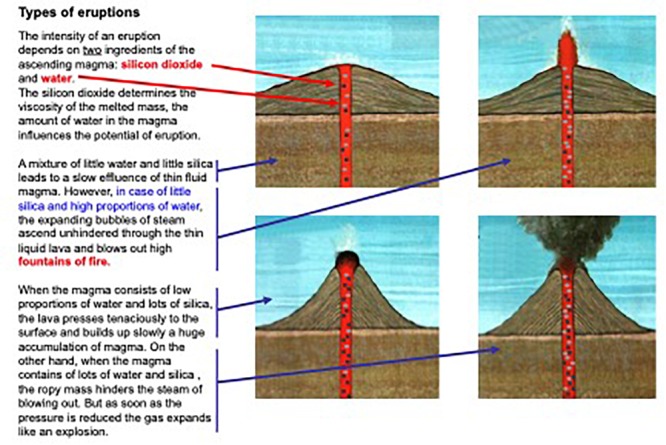
Example of the training material with highlighted element-to-element and relation-to-relation mappings.

Each step is formulated as a task or a question that has to be answered, e.g., what are the relevant data points in the diagram or what does the text explain that cannot be seen in the picture. After having read the introduction of each strategy the workbook provided a worked example for either a text, a picture or a text-picture combination, where the different steps of the strategy were implemented and annotated (like in the study of Berthold et al., 2007). Only then learners were asked to apply the strategy on their own with a new text, picture or text-picture combination. The training materials were all in the domain of natural science but in different areas, like geography, biology or ecology (for an example see Figure 1). The worked examples were always in a different scientific area than the practicing examples.

After this session, participants of the experimental group exercised the strategies by using the workbook for 3 weeks. In the 1st week they practiced the text reading strategy, in the second the picture reading strategy and in the 3rd week the mapping strategy with texts and pictures. Participants were reminded via email. They had to apply the strategies by using representations from their daily live or from current lessons of their study program. With this, we intended to provide a more meaningful setting and thus to enhance compliance and strategy transfer. We collected participants practicing materials every week at the beginning of the seminar course and checked for traces of strategy use. We found clear evidence for strategy use in all texts, pictures and text-picture-combinations.

#### Alternative Program for the Control Group

The treatment of the control group also comprised a 90-min session on-site and a 3-week elaboration phase outside the classroom. During the seminar session, students had to work on the pros and cons of the use of new media in school. First, students had to read introducing texts and discussed them afterwards in a teamwork discussion during the seminar. Subsequently, they further discussed this issue in a 3-week lasting online discussion forum. Thus, the topic was not related in any way to strategies for reading or integrating texts and pictures. Both groups had a 3 weeks period to work on their tasks. The material that was handed in by the training group as well as the statements in the discussion forum indicates that they all spent a reasonable amount of time. Due to ethical reasons the control group also received the strategy training material after the last session. The learning material for the experimental as well as for the control group can be seen in the Supplementary Datasheets S1, S2.

#### Posttest Session

In the posttest session (at the end of the 3-week training or online-discussion session) both groups received learning material about the function of a four-stroke Otto engine. The experimental group was instructed to use the acquired learning strategies whereas the control group had no further instruction besides the task description. The material comprised a brief introduction to the function of the Otto engine and a labeled picture of the engine’s structure. The processes of the 4 strokes were nevertheless only described verbally while the four pictures of each of the four strokes were given unordered at the end of the learning material. While studying the material, learners had to relate the appropriate picture to the corresponding description of each stroke. The number of the correct relations indicated global coherence formation and was included in the comprehension measure of the post-test that was conducted after learning. The test comprised 3 open-ended recall tasks, asking for the most important propositions of the text. In addition, learners had to sketch the picture with its labels. Comprehension was measured with 3 open questions where learners had to draw inferences from what they learned from the text and picture. In addition, the score of the text-picture relation task was integrated. The recall test had a maximum score of 16.5 points, 11 points could be reached in the comprehension test. Cronbach’s Alpha was sufficient for the recall measure (α = 0.74) but lower for the comprehension test (α = 0.63) due to its various inference tasks.

## Results

### Descriptive Results

As can be seen in Table 2, Learners’ prior knowledge was overall on a very low level and their spatial abilities as well as their working memory capacity were on a medium level. A MANOVA with the treatment (training versus no training) as independent variable and prior knowledge, spatial ability and working memory capacity as dependent variables revealed that the groups did not differ concerning their prior knowledge, and their spatial abilities (*F*s < 1), ns and also not significantly concerning their working memory capacity [*F*(1,28) = 2.22, *p* = 0.15]. However, the Kolmogorov–Smirnov-Test revealed that prior knowledge was not normally distributed, [*D*(28) = 0.22, *p* < 0.01], but the Levene-test showed homogeneous variances as well (*F* < 1, ns). Thus, the results have to be interpreted carefully, mainly based on a descriptive level.

**Table 2:** Means (standard deviations) for control variables and dependent variables in both groups.

	No Training (*n* = 15)	Training (*n* = 15)
Prior knowledge (maximum 11.5)	2.87 (3.55)	2.17 (2.96)
Spatial abilities (%)	68.76 (17.84)	67.88 (13.31)
Working memory capacity	4.00 (1.41)	3.27 (1.28)
Recall (maximum 16.5)	8.70 (3.87)	9.20 (3.90)
Comprehension (maximum 11)	6.13 (2.80)	6.47 (2.97)

As both control variables, i.e., spatial ability and working memory capacity were positively correlated with recall (*r*_spatial_ = 0.52, *p* < 0.01; *r*_wmc_ = 0.31, *p* < 0.10) and comprehension measures (*r*_spatial_ = 0.50, *p* < 0.01; *r*_wmc_ = 0.44, *p* < 0.05) we entered them as covariates in the subsequent analyses.

### Treatment Effects in Interaction With Learners’ Prior Knowledge

In order to test the hypotheses whether the training is effective compared to no training and whether these effects depend on learners’ prior knowledge a regression model was analyzed for recall and comprehension performance with the following predictors: treatment, prior knowledge, the product term treatment ^∗^ prior knowledge, working memory capacity and spatial abilities. At first, treatment was coded with 0 for the control group and 1 for the training group. In a second step the treatment factor was recoded (control = 1, training = 0) and the regression analysis was conducted again. With this method of “recentering”, proposed by Aiken and West (1991), it is possible to analyze the specific impact of prior knowledge for the respective group which is coded with 0 as reference group. First, it has to be noted that the Kolmogorov–Smirnov-Test revealed that the recall data were normally distributed [*D*(28) = 0.11, *p* > 0.05], but that the comprehension scores were not [*D*(28) = 0.173, *p* < 0.05]. In addition the Levene-test revealed that the variances for both outcomes measures were homogeneous [recall: *F*(1,28) = 4.06, *p* > 0.05; comprehension: *F*(1,28) = 1.142, *p* > 0.05].

**FIGURE 2:**
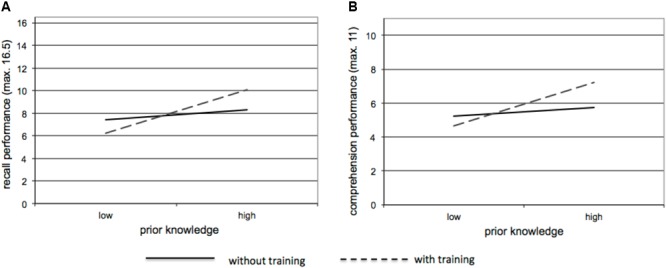
Regression slopes for the impact of prior knowledge per group regarding recall **(A)** and comprehension performance **(B)**.

For *recall* performance the regression model was significant, [*F*(5,29) = 6.25, *p* = 0.001, *R*^2^adj = 0.48]. The treatment factor (training versus no training) was not significant [beta = 0.43, *t*(29) = 0.26, *p* = 0.79]. Learners in the two groups showed almost the same performance (for all outcome measures see Table 2). However, the aptitude-treatment-interaction was significant [beta = 0.42, *t*(29) = 2.17, *p* = 0.04]. Thus, the influence of learners’ prior knowledge differed significantly between the groups, as Figure 2A depicts: while prior knowledge had no influence in the CG [beta = 0.19, *t*(29) = 1.04, *p* = 0.31] it had a significant influence in the EG [beta = 0.81, *t*(29) = 3.72, *p* = 0.001]. With increasing prior knowledge learners showed increased learning performance in the training group. Spatial abilities also turned out to be significantly predictive [beta = 0.37, *t*(29) = 2.46, *p* = 0.02]. Working memory capacity had a positive but non-significant influence [beta = 0.30, *t*(29) = 1.89, *p* = 0.07].

For *comprehension* performance we found almost similar results (see Figure 2B). The overall model was significant [*F*(5,29) = 6.18, *p* = 0.001, *R*^2^adj = 0.47]. The training had no overall effect [beta = 0.08, *t*(29) = 0.51, *p* = 0.61]. The interaction pattern is also significant [beta = -0.48, *t*(29) = -2.04, *p* = 0.05]. Again, the differentiated analyses revealed that prior knowledge had no influence in the CG [beta = 0.15, *t*(29) = 0.81, *p* = 0.43] but significantly predicted comprehension performance in the EG [beta = 0.73, *t*(29) = 3.35, *p* = 0.003]. Again learners with increasing prior knowledge revealed higher comprehension scores in the training group. Comprehension was not significantly influenced by spatial abilities [beta = 0.30, *t*(29) = 1.99, *p* = 0.06] but by working memory capacity [beta = 0.46, *t*(29) = 2.90, *p* = 0.008].

## Summary and Discussion

Texts are often enriched with pictures and based on the well-known multimedia principle learners can profit from such a combination (Butcher, 2014). However, the beneficial effects of an additional picture only pay off when learners actually integrate text and picture information into one coherent mental representation (Ainsworth, 2006). In this study a training was developed and analyzed that provides learners with the crucial steps of understanding and integrating text and picture combinations. Overall, it was assumed that the training could be helpful for learning but that these effects will be moderated particularly by learner’s prior knowledge.

In fact, we found no overall positive effect of the training, neither for recall performance nor for comprehension performance. Thus, the training is not effective in general.

However, we could confirm the expected moderating effect of prior knowledge for *recall performance*. The first and main result is that prior knowledge especially affected the results in the training condition. As assumed, learners could only profit from the training with sufficient prior knowledge, i.e., we found an enhancing effect of prior knowledge. With insufficient prior knowledge the training was not effective or even hindered learning. These findings are in line with previous studies on situational help for coherence formation (Seufert, 2003; Seufert et al., 2007). They also found that help is only effective for learners with sufficient but not too high levels of prior knowledge. These learners still are in need of help and are capable of using it. In our sample only 15% of the learners reached at least half of the possible scores in the pre-test, thus we only have very few learners with higher expertise. So we can ask how experts would have performed with the training. Based on the expertise reversal effect (Kalyuga, 2007) where expert learners are actually hindered by unnecessary help, one could assume that our training may also produce such reversal. Experts would not need the strategy, because they can extract the semantic structure of text and picture based on their knowledge. Moreover, the proposed strategy might even interfere with their existing strategies and has to be ignored actively causing unnecessary burden on learners’ resources.

The second interesting aspect of the interaction pattern we found is that prior knowledge has no significant influence on recall in the control condition. Without any further help even higher knowledgeable learners show only medium performance scores. This is further evidence for the argument that many learners have substantial difficulties in integrating text and picture and that assistance is needed (see Ainsworth, 2006; Renkl and Scheiter, 2017). Nevertheless, there is further evidence needed with a greater sample with normally distributed scores of learners’ prior knowledge. Until then, the results should be interpreted carefully mainly based on a descriptive level, that shows the different slopes of the two groups.

Concerning the effects of the training on *comprehension performance* we also found an influence of learners’ prior knowledge, but with a smaller effect. We again found the same pattern that with increasing prior knowledge learners profited from the training and once more prior knowledge showed no effect in the no training group. Again, one has to consider the effects with care as the prior knowledge scores as well as the comprehension scores were not normally distributed. Nevertheless, the slopes show different increases but we would have expected even stronger effects on comprehension performance as the training explicitly aimed at semantic mapping processes. And especially when it comes to comprehension, learners should profit from their prior knowledge as this could help to link new and existing knowledge and to build meaningful schemata. One could speculate why learner’s prior knowledge does not have the expected stronger enhancing function while using the trained strategy for comprehension. Maybe learners do not make the link between their existing knowledge and the new information or they do not aim at understanding the material even with prior knowledge. Instead they might integrate on a surface level by syntactic mappings. When taking a closer look at the structure of the strategy training, it is also plausible that learners tend to follow the strategy instructions stepwise in a successive order. Thus, they first primarily focus on elements and relations in the text, then on the picture and only afterwards they link both structures. With these fine-grained analyses of the two sources, the overall picture might get lost and learners do not strive at building an overall network of all the information where they could effectively use their existing network of knowledge.

But all these possibilities remain speculative since we have no further indicators for the processes learners actually execute. Process data like thinking aloud protocols or eye-tracking data could provide further information about if and how the strategy is applied, whether it needs to be refined or whether additional help is needed. One could also learn more about the interplay with learners’ prior knowledge. Despite the processes, it would also be valuable to analyze not only cognitive but also motivational effects of the training. The effects of the training will surely depend on the commitment the learners have toward the strategy and this in turn surely depends on whether they actually evaluate it as useful. Seufert (2018) suggests that the amount of regulation, in our case the intensity of using the trained strategy, depends on the necessity of this strategy to accomplish the goal, the available resources to accomplish the strategy and the resulting load imposed by the strategy use. While we analyzed learner’s prior knowledge as one crucial resource, we did not take into account the necessity or the appraisal of usefulness as suggested above. Additionally, we did not investigate the experienced load when using the strategy. As argued above the use of a newly trained strategy could impose additional load in terms of extraneous load as it is not yet automated and requires resources for conducting and monitoring the proceeding steps. In contrast, one could also assume that strategy assistance could also relieve learners as they are guided step by step. The study of de Bruin et al. (2005) provides evidence for such a relieving effect of a strategy instruction. However, as the strategy in our study was very complex and surely cannot be automatized after only 1 h or even 3 weeks of occasional exercises, we would assume an increase in cognitive load. Moreover, we additionally asked learners to reflect on their strategy what could impose an additional metacognitive load (Bannert, 2002). In terms of germane processing one could also assume that learners who are able and willing to follow the strategy would also invest germane resources. Thus, a differentiated measurement of learners’ perceived extraneous and germane load could further enlighten the actual effects of the training (Klepsch et al., 2017).

Another important point is that the training should be compared to a stronger control condition, which also provides a strategy training, like e.g., on metacognition. In both groups learners would then have to handle an additional strategy while learning and hence the cognitive affordances would be comparable. Only then one could qualify the effects on learning outcomes as effects of a training on coherence formation in contrast to an alternative training.

Overall, we developed a training that can be helpful for coherence formation if learners have sufficient prior knowledge and are thus able to deal with the possible additional burden and therefore can accomplish the strategy successfully. With this constraint one cannot actually recommend to implement the strategy training in instructional settings. To ensure that also learners with low prior knowledge can benefit from strategy instruction the affordances have to be further decreased. This could be accomplished by either providing pre-training where the most relevant concepts of the learning domain are conveyed or by segmenting the elements of the coherence formation strategy (Ayres, 2013). Learners could first be provided with strategies for single representations like text or reading strategies. Only when these strategies are automated the next level of coherence formation should be addressed. Segmenting could lessen the intrinsic cognitive load – in this case of the strategy – and therefore even novices could learn successfully. Moreover, with an extended research program with variations of the training, it would also be possible to analyze differential effects of the training components. Are all training components necessary, which of them produce the strongest effects, for which processes and for whom? Hence, it would be interesting and necessary to take a deeper insight into the learner’s mind by asking them to think aloud or to evaluate their load repeatedly and differentially. In spite of a successive implementation of the strategy parts one could also think of a fading strategy to ensure that learners are able to conduct the strategy autonomously. Studies on fixed versus faded prompts show promising effects on strategy transfer in the long run (Davis, 2003). Generally, it could be interesting to introduce a follow-up measure to see whether the positive effects for high prior knowledge learners persist or whether there are any sleeper effects for low prior knowledge learners: it would be possible that the trained strategies are practiced in the meantime, so that they can be carried out in the follow-up test with less mental effort, resulting in improved learning outcomes. If this were the case, we would have a strong argument for enlarged training programs, which could be implemented in classroom teaching over a longer period of time.

However, even if the study provides some first insights in how and for whom strategies for text-picture integration can be trained it also has some major shortcomings. The major problem is the very small sample size that hampers a broad generalizability and restricts the statistical power. In addition, the sample also mainly consist of low prior knowledge learners. Thus, we could not ensure normal distribution of the data. In the naturalistic setting (with the training being part of a whole course with repeated training or testing phases), which was chosen to ensure the external validity of the study, it was not possible to obtain a greater number of participants with less skewed data. The complex procedure with high affordances for the students’ commitment can be seen as an additional flaw. Whether participants’ commitment was actually high cannot be ensured, but at least it should have been assessed in an appropriate way. This could have helped to qualify the intensity of strategy use. Also the students’ products when using the strategy with their own study materials in the 3 weeks after the strategy training session could have been analyzed. However, as they are further needed in their courses they could not hand them over. For replicating the effects of the training one should ensure a larger sample in a classroom setting over a longer period of time where one could implement the strategy as inherent part of the curriculum. This could allow a deeper insight in the processes and products and more complex analyses of mediating or moderating effects. Based on these, one could refine the training and might even find an adaptation mechanism to ensure effective trainings for learners based on their individual learner characteristics and on their individual progress.

## Ethics Statement

This study was exempt from an ethic committee approval due to the recommendations of the German Research Association: All subjects were in no risk out of physical or emotional pressure, we fully informed all subjects about the goals and process of this study and none of the subjects were patients, minors or persons with disabilities. Participation was voluntary and all subjects signed a written informed consent and were aware that they had the chance to withdraw their data at any point of the study.

## Author Contributions

The author confirms being the sole contributor of this work and has approved it for publication.

## Conflict of Interest Statement

The author declares that the research was conducted in the absence of any commercial or financial relationships that could be construed as a potential conflict of interest.
