# Supplementary material for: Training for Coherence Formation When Learning From Text and Picture and the Interplay With Learners’ Prior Knowledge
Source: Front Psychol. 2019 Feb 7;10:193. doi: 10.3389/fpsyg.2019.00193 (PMC6374309; doi:10.3389/fpsyg.2019.00193)
Supplement: Supplementary file 1 [file Data_Sheet_1.PDF]

Successful strategies
for multimedia learning

an experimental study in the context of the seminar
„Learning with media"
Dr. Tina Seufert

Dear students,
You agreed to participate in our experiment on multimedia learning, thank you very much!

The goal of this workbook is to provide you with some strategies that could ease your use of multimedia learning material. Please carefully work with the textbook for the next 90 minutes.

The single sections will be explained in detail. If you have any questions do not hesitate to ask the investigator.

At the end of the experiment we would like to collect your textbooks with your notes. Therefore we ask you to use your code, so that we can use the data anonymously:

Code ___________________

Enjoy!

What's the study about?

During the last years multimedia learning material got increasingly important. On the one hand this has big advantages, as leaning content can be presented dynamically and interactively. On the other hand it could been shown that earning is more diversified but not always successful. The reason for that is that many learners lack effective strategies for dealing with multimedia learning material.

In our study we want to show you such strategies. Thereby we will concentrate on the two main forms, that can be found in almost all multimedia learning materials but also in traditional paper-based materials, namely texts and pictures!

The textbook comprises three sections:

1. The first section shows you good strategies for reading text.
2. In the second section we will show you strategies to better understand pictures
3. In the third section we will provide you with strategies for intensively working with text and pictures that are presented in combination, so that you can better understand their relation.

For each section you will find a summarizing strategy-sheet which is printed on colored paper. Thus, you can easily find the strategy instruction whenever you need it.

Text comprehension strategy – the 5-steps-reading method

Almost every learning material includes text. Therefore it is important to have effective strategies for dealing with text. Surely you have a lot of experience with texts and will be able to grasp the most important issues in it. But are you also able to memorize what you have read in the long run?

In the following we want to give you some hints how to systematically and intensively work with texts, in order to grasp the crucial information and to keep them longer in your memory.

Get a first overview

First you should get an overview of the text. What is the text about? Trying to "scan" the structure of the text: the headlines, highlighted words, the beginnings of sections

Think of what you already know

Think of all that you already know about the issue of the text. Do you have any associations? Make notes in bullet-style form.

Read

Now read the text carefully. Underline the most important terms (Not to many!)

Structuring

After the first reading you should go through the text once again. Do you recognize meaningful sections? Mark them at the margin. Summarize each section the short note at the margin. You can also underline one statement within each section, but use a different color as those you used for marking important terms.

Summarize / link to your memory

To better memorize the main assumptions of the text it is important that you link it's to your prior knowledge and to structure the new information meaningfully.

Therefore, go back to the notes he first made on your prior knowledge. Can you link them to what you just read in the text?
Summarize the main assumptions of the text your own words in max. five sentences. Try to link it to your prior knowledge. You can also sketch a picture or diagram that summarizes the most important statements of the text. Bottles of the picture in the diagram should express the relation to your prior knowledge.

No we want to show you this strategy with a short text example….

Types of eruptions

The **intensity of an eruption** depends on two ingredients of the ascending magma: **silicon dioxide** and **water**.
The silicon dioxide determines the **viscosity** of the melted mass, the amount of water in the magma influences the **potential of eruption.**

A mixture of little water and little silica leads to a **slow effluence** of thin fluid magma. However, in case of little silica and high proportions of water, the expanding bubbles of steam ascend unhindered through the thin liquid lava and blows out high **fountains of fire.**

When the magma consists of low proportions of water and lots of silica, the lava presses tenaciously to the surface and builds up slowly a **huge accumulation of magma**. On the other hand, when the magma contains of lots of water and silica , the ropy mass hinders the steam of blowing out. But as soon as the pressure is reduced the **gas expands like an explosion.**

1. Overview

2. prior knowledge?

I can remember that I have already seen expanding vulcanos . They actually looked quite different.

3. Reading

Mark terms (s. the text)

4. Structuring

Make notes at the margin (see below)

5. Summarizing

Depending on the composition of the magma there are different types of eruptions:
Water is responsible for exploding powers (that reminds me of a pressure cooker)

silicon dioxide is responsible for the solidity of the lava (by the way that's also the case for finger nails)

example for the 4th step – the structuring…

Types of eruptions

The **intensity of an eruption** depends on two ingredients of the ascending magma: **silicon dioxide** and **water**.
The silicon dioxide determines the **viscosity** of the melted mass, the amount of water in the magma influences the **potential of eruption.**

2 important ingredients:

1) silicone dioxide ➡ viscosity

2) water ➡ erupting potential

A mixture of little water and little silica leads to a **slow effluence** of thin fluid magma. However, in case of little silica and high proportions of water, the expanding bubbles of steam ascend unhindered through the thin liquid lava and blows out high **fountains of fire.**

When the magma consists of low proportions of water and lots of silica, the lava presses tenacously to the surface and builds up slowly a **huge accumulation of magma**. On the other hand, when the magma contains of lots of water and silica , the ropy mass hinders the steam of blowing out. But as soon as the pressure is reduced the **gas expands like an explosion.**

4 possible typen:

1) few / few	➡	slow effluence
2) few / much	➡	fire fointain
3) much/ few	➡	accumulation
4) much / much	➡	heavy explosion

Exercise for the text comprehension strategy

On the following pages you will find a text for practicing the 5-steps-reading method on your own. Use the instruction for the strategy on the colored page.
Also use colored pens and do not hesitate to make notes or underline terms and passages in the text book. Additional paper can be found in front at the investigator's desk.

example text, e.g. from wikpedia, about 1-2 pages long (with only few or no pictures)

Picture comprehension strategy – The 3-W-Method

What would learning materials look like without pictures. They visualize the content and show the main aspects and relations with one view. Most times we find pictures more attractive and more motivating than texts. Neverhless, we very often only look briefly on them so that their main message does not reach us. We now want to provide some hints that could help to take a closer look at pictures.

Therefore the following three W-questions can be helpful:
1. Which type of picture is this?
2. What do I see?
3. What does the author of the pictures wants to tell me?

1. W-question: Which type of picture is this?

The right view on a picture starts with the choice of the right lenses:
What type of picture is this? In general we can differentiate between…

Realistic pictures, e.g.

- Line drawings
- Paintings
- Photographs
- Comics
- Maps

Logical pictures, e.g.

- Structural diagrams
- Pie graph
- Bar graph
- Line Graph
- Scatter plot
- mind maps

Examples for both types of pictures can be found on page 9.

2. W-question: what do I see?

Realistic picture
- How abstract/ realistic is the picture?
- What are the crucial picture parts? (figure-ground-differentiation)
  - Use highlights
  - Use shadows or colors as information
- Can some Picture parts can be grouped?
- Can larger picture parts be separated

Logical picture
- Which type of diagram is this?
- Do I know this type of diagram?
- What elements usually belong to this type of diagram? Are these elements included?

- E.g scatter plot, line- or bar diagram:
  - Coordinate (axes, scales of the axes)?

    - Labels of the axis
    - Points, bars, Lines, curvel
    - Legends
- E.g. Pie graph
    - Which pie segments are there?
    - Are they labeled?
    - Is the angle size explicative or can it be guessed?
- E.g. Structural diagram / mind map
    - What kind of structure do I see? (hierarchic, linear, network)
    - Are there central elements?

3. W-Question: what does the author of the picture wants to tell me?

Realistic picture

- Is there a picture heading or any information on the picture in the text?
- What do I already know about the content?
- Which graphic elements are central? (color them if possible)
- What does the author wants to tell me with this picture?
  Summarize the main statement(s) of the picture and try to link it with your prior knowledge.

Logical pictures

- Is there a picture headline or any information on the picture in the text?
- What do I already know about the content?
- What does the graphic elements mean?
  For this, labels of axes, headlines or legends could be helpful Which variables are presented? What is the range of these variables?
- When you read out information from a picture there are three different levels (see the example picture on the amount of scoops the kids eat):
    - Reading 1st order:
      single information is extracted, like single points in a scatter plot or the height of one bar in a bar graph
    - Reading 2nd order:
      two singular elements are extracted and compared, like two point two bars or the gradient of a line (increasing or decreasing)
    - Reading 3rd order:
      two pairs of information are compared like the course of two lines
- What is the main message of the picture? Summarize the main statement(s) of the picture and try to link it with your prior knowledge.

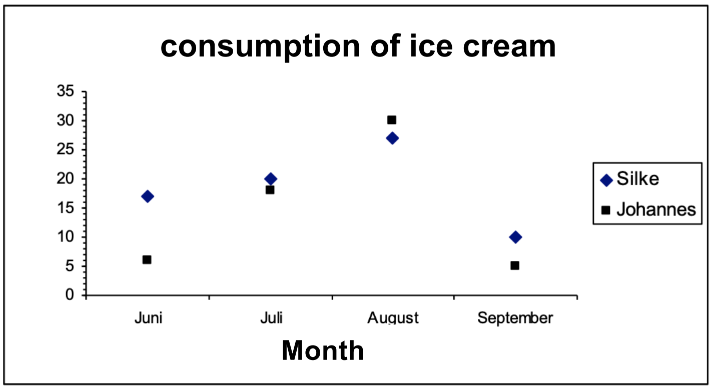

Reading 1st order:
Silke ate 17 scoops in June

Reading 2nd order:
Johannes ate more ice cream than Silke in August

Reading 3.rd order:
Johannes' consumption of ice cream increases stronger than Silkes' until August and also decreases mores steeply afterwards

Examples for realistic pictures

[Example pictures like the following]

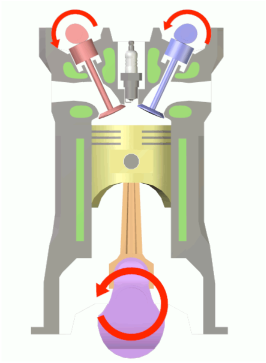

https://commons.wikimedia.org/wiki/File:Four_stroke_cycle_start.png

Examples for logical pictures

[Example pictures like the following]

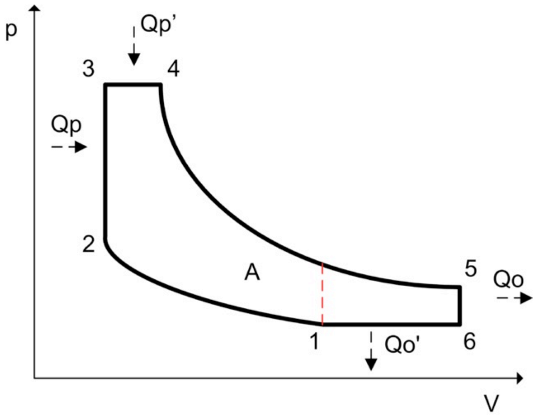

https://commons.wikimedia.org/wiki/File:T_cycle_AtkinsonMiller.png

Now you will find two examples for the use of the 3-W-method in picture reading

Example 1

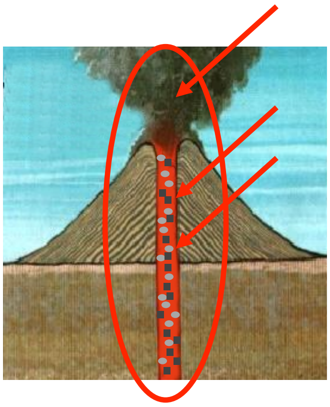

1. What type of picture?

realistic picture

2. What do I see?

abstract /realistic? the picture s rather abstract

figure-ground-differentiation: the volcano is central, sky and ground are in the background

grouping picture parts: hill, ash cloud and the red stripe with the dots belong together.

separate picture parts: does not seem to be applicable here.

3. What does the picture wants to tell?

picture headline? Info in text? not existing

prior knowledge? looks like an eruption to me. Earlier I've read that water and silicon oxide are responsible for the the type of explosion. Maybe this has something to do with the grey and black dots? the red stripe reminds me of lava or magma.

mark crucial elements (see the highlights in the picture)

summary:
the picture shows the eruption of a volcano and it seems that the amount of grey and black dots has something to do with the type of explosion. When I link it to what I've read before, there could be much water, because the eruption looks heavy. How the lava looks like I cannot really see.

Example 2

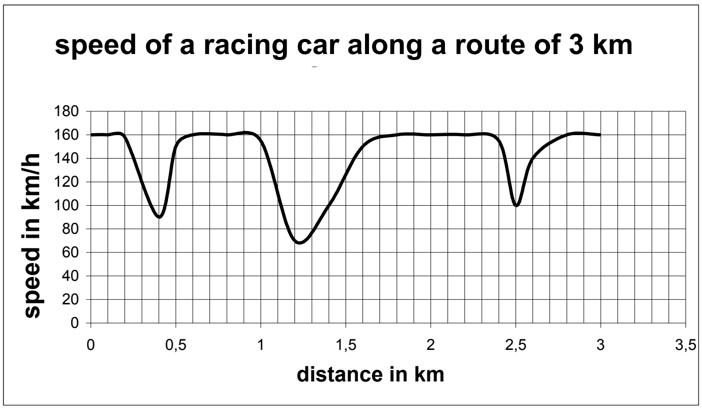

1. What type of picture?

logical picture

2. What do I see?

which type of diagram? curve chart

Do I know this type of diagram? Yes

What belongs to it? coordinates (axes with labels), curves/lines, legend

Are these elements there? yes both axes are labelled and have a scale. a legend is missing but not necessary because there is only one curve.

3. What does the picture wants to tell?

picture headline /info in text? There is a headline.

What does the graphical elements mean? The curve shows the progress of speed in km/h on a route of 3 km length.

Which variables are presented (range)? the first variable is speed, varying from 0 to 180 km/h, the second variable is the distance from 0 to 3 km.

Reading: 1st order: e.g. the slowest point is at 1,2 km; 2nd order: three times the car slows down and accelerates again afterwards to 160 km/h

What is the main message of the picture? a racing progress is shown that seems to have three curves or obstacles. The car can drive 160 km/h at most. I do not have any prior knowledge on that issue.

On the following pages you will now find two examples with which you can exercise the 3-W-method for yourself. Use the instruction of the 3-W-method on the colored page

Example picture 1

[Example picture with a plant cell and an animal cell in comparison side by side]

Example picture 2

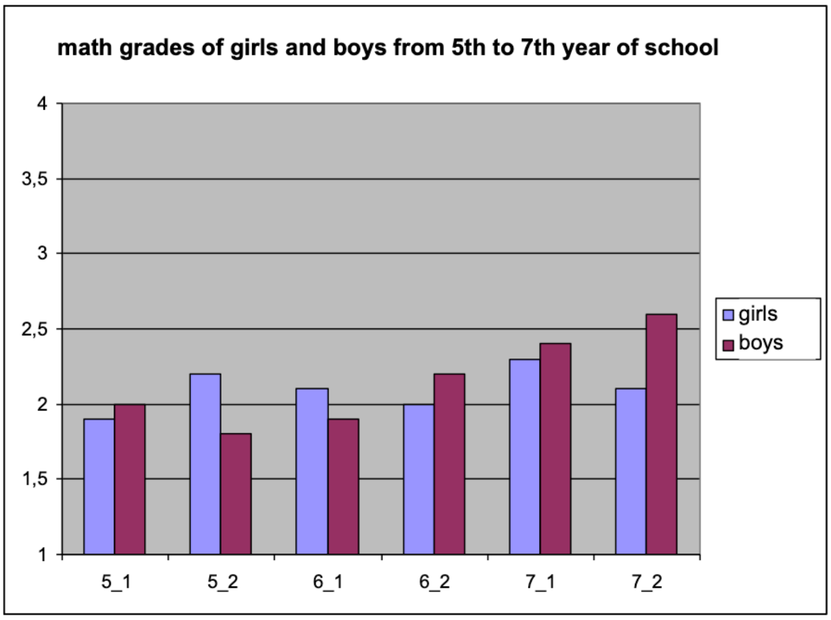

Strategies to meaningfully link text and picture

In most learning materials you can find combinations of a text and a picture that together explain an issue. Thus, it is important to not only intensively process text and picture alone but also to understand their relation.
Sometimes this linking opens new perspectives on the text / the picture, which one could miss when text or picture are processed isolated. Moreover, pictures often help to better understand the text or vice versa. It even eases understanding if one sees text and picture as one unit, which is thus linked together while "reading".

The principle of this linking is very simle – we call it MAPPING.
The English word „mapping" means to map things onto each other. This process comprises 4 steps when we map text and picture onto each other (see also the schematic picture):

1. Elements that are included in the text as well as in the picture are mapped onto each other, thus they are linked. The first step is therefore called e*lement-to-element-mapping.*
2. If there are statements/relations in the text which are also presented in the picture, these statements should also be mapped onto each other. This second step is therefore called *relation-to-relation-mapping.*
   For mapping it is important to link corresponding information. However, this does not mean that the mapped information is redundant: maybe the text makes a statement to an element, which can be also seen in the picture. But this statement might be more elaborated than what can be seen in the picture. Or relations of elements in the picture complement the corresponding elements in the text.
3. Besides these complementary information texts usually also comprise elements and relations that are not presented in the text. The same is the case for the picture. Thus, the third step is to find this *complementary information*.
4. In the last step one should work out the overall message of text and picture. Together, corresponding and complementary information form a network of information that could be explicated in a few sentences or by creating a structural diagram. Again, you should try to link the overall information to your prior knowledge.

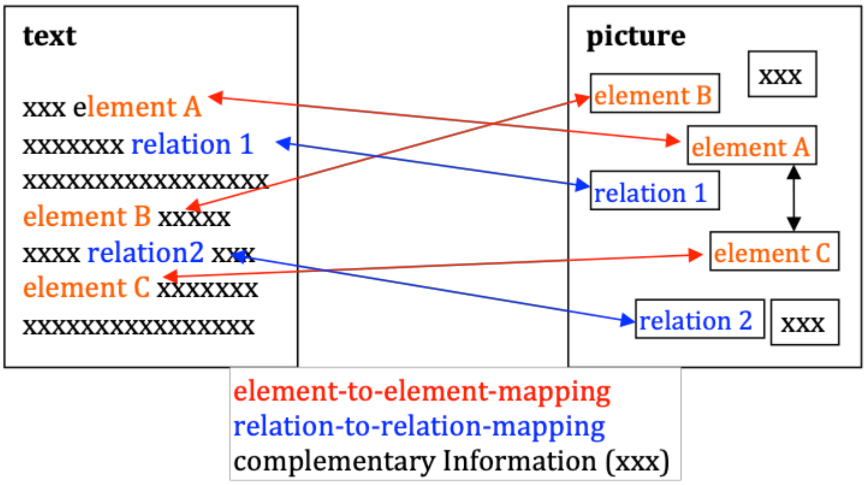

On the next page we summarized all the steps that are necessary, when linking text and picture.

Checklist for linking text and picture

Scanning

▪ read the headline of the text<br>▪ „scan“ the picture	➡ What do I already know about the issue?<br>➡ Do text headline and picture fit together?

Read the text

▪ get an overview<br>▪ activate prior knowledge<br>▪ reading<br>▪ structuring<br>▪ summarizing	➡ mark crucial elements<br>➡ make notes on main messages/relations at the margin

Read the picture

▪ What type of picture is it?<br>▪ What do I see?<br>▪ What does the picture wants to tell?	➡ mark crucial elements<br>➡ make notes on main messages/relations at the margin

Mapping

▪ element-to-element-mapping<br>▪ relation-to-relation-mapping<br>▪ complementary Information (xxx)	➡ mark corresponding elements (e.g. link them with arrows or mark them in the same color)<br>➡ mark corresponding relations (arrows, color)

Explicate the overall message

▪ What is the overall message of text and picture?<br>▪ How does this message link to my prior knowledge?	➡ Summarize the main message of both, text and picture in 4-5 sentences or in a structural diagram/mind map<br>➡ Thereby, explicate the link to your prior knowledge

What is the relation between text and picture?

- are they redundant?
- are they independent from each other?
- is one of them dispensable?

On the next pages we will show you a detailed example of how to use the text-picture-mapping strategy before you can start to exercise the strategy yourself.

1. Scanning

What do I already know about the strategy?

I already know the text and one of the pictures (the lower right one).

Do text headline and picture fit together?

Yes, they both describe different types of eruptions and the picture shows how the 4 types look like.

Types of eruptions

The intensity of an eruption depends on two ingredients of the ascending magma: silicon dioxide and water.
The silicon dioxide determines the viscosity of the melted mass, the amount of water in the magma influences the potential of eruption.

A mixture of little water and little silica leads to a slow effluence of thin fluid magma. However, in case of little silica and high proportions of water, the expanding bubbles of steam ascend unhindered through the thin liquid lava and blows out high fountains of fire.

When the magma consists of low proportions of water and lots of silica, the lava presses tenacously to the surface and builds up slowly a huge accumulation of magma. On the other hand, when the magma contains of lots of water and silica , the ropy mass hinders the steam of blowing out. But as soon as the pressure is reduced the gas expands like an explosion.

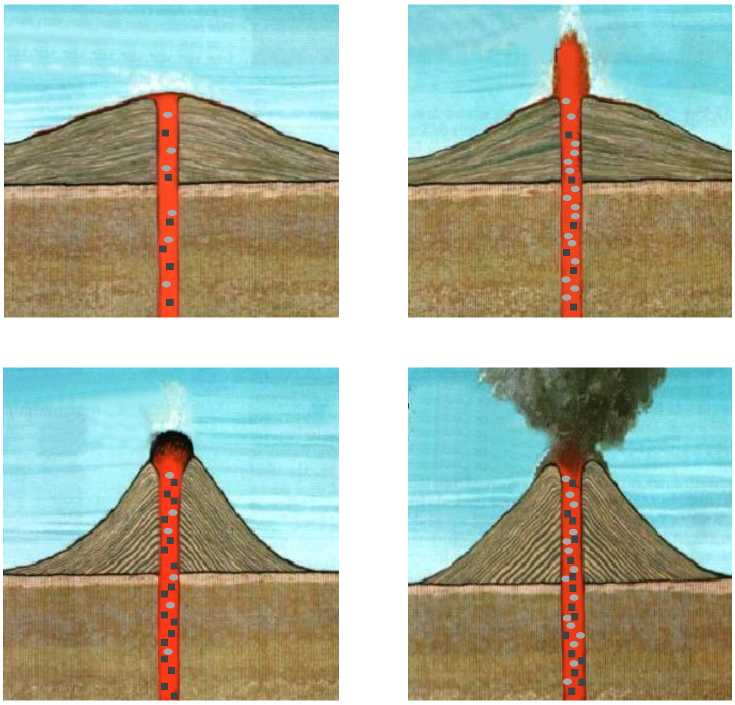

4 different representations => types of eruptions

2. Read the text

Mark crucial elements
Make notes on main messages/relations at the margin

Types of eruptions

The **intensity of an eruption** depends on two ingredients of the ascending magma: **silicon dioxide** and **water**.
The silicon dioxide determines the **viscosity** of the melted mass, the amount of water in the magma influences the **potential of eruption.**

2 important ingredients:
1) silicone dioxide ➡ viscosity
2) water ➡ erupting potential

A mixture of little water and little silica leads to a **slow effluence** of thin fluid magma. However, in case of little silica and high proportions of water, the expanding bubbles of steam ascend unhindered through the thin liquid lava and blows out high **fountains of fire.**

When the magma consists of low proportions of water and lots of silica, the lava presses tenacously to the surface and builds up slowly a **huge accumulation of magma**. On the other hand, when the magma contains of lots of water and silica , the ropy mass hinders the steam of blowing out. But as soon as the pressure is reduced the **gas expands like an explosion.**

4 possible typen:

1) few / few	➡	slow effluence
2) few / much	➡	fire fointain
3) much/ few	➡	accumulation
4) much / much	➡	heavy explosion

3. Read the picture

Mark crucial elements
Make notes on main messages/relations at the margin

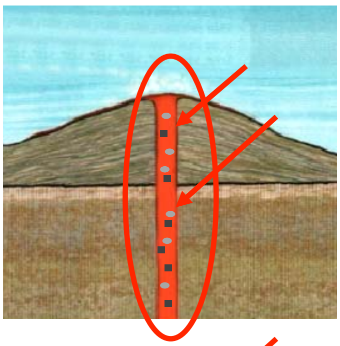

few ■ and few ●

leads to weak eruption
(with a flat hill)

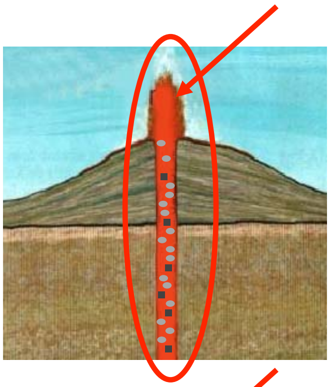

few ■ und much ●

leads to a heavy eruption
with fire
(with a flat hill)

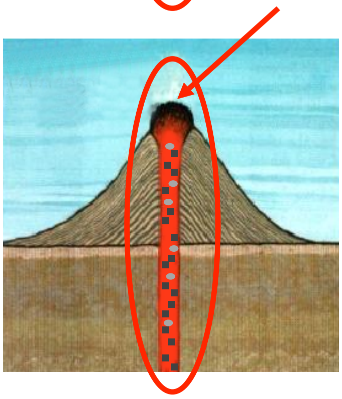

much ■ und few ●

leads to
clot building
(with a steep hill)

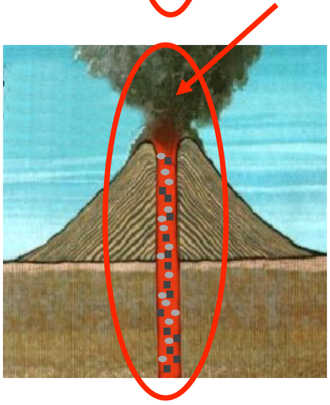

much ■ und much ●

leads to a heavy
eruption with smoke
(with a steep hill)

4. Mapping

Mark corresponding elements
Mark corresponding relations

Types of eruptions

The intensity of an eruption depends on two ingredients of the ascending magma: **silicon dioxide** and **water**.
The silicon dioxide determines the viscosity of the melted mass, the amount of water in the magma influences the potential of eruption.

A mixture of little water and little silica leads to a slow effluence of thin fluid magma. However, in case of little silica and high proportions of water, the expanding bubbles of steam ascend unhindered through the thin liquid lava and blows out high **fountains of fire.**

When the magma consists of low proportions of water and lots of silica, the lava presses tenaciously to the surface and builds up slowly a huge accumulation of magma. On the other hand, when the magma contains of lots of water and silica , the ropy mass hinders the steam of blowing out. But as soon as the pressure is reduced the gas expands like an explosion.

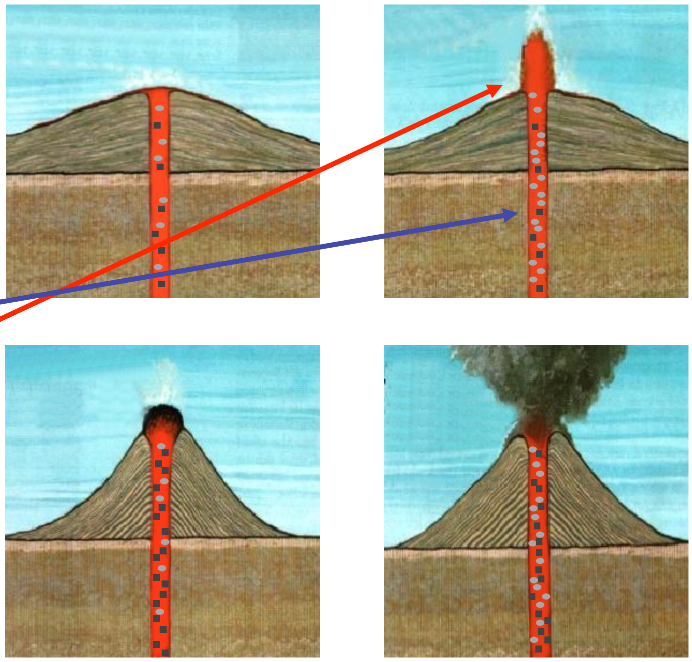

Types of eruptions

The intensity of an eruption depends on two ingredients of the ascending magma: **silicon dioxide** and **water**.
The silicon dioxide determines the viscosity of the melted mass, the amount of water in the magma influences the potential of eruption.

A mixture of little water and little silica leads to a slow effluence of thin fluid magma. However, in case of little silica and high proportions of water, the expanding bubbles of steam ascend unhindered through the thin liquid lava and blows out high **fountains of fire.**

When the magma consists of low proportions of water and lots of silica, the lava presses tenaciously to the surface and builds up slowly a huge accumulation of magma. On the other hand, when the magma contains of lots of water and silica , the ropy mass hinders the steam of blowing out. But as soon as the pressure is reduced the gas expands like an explosion.

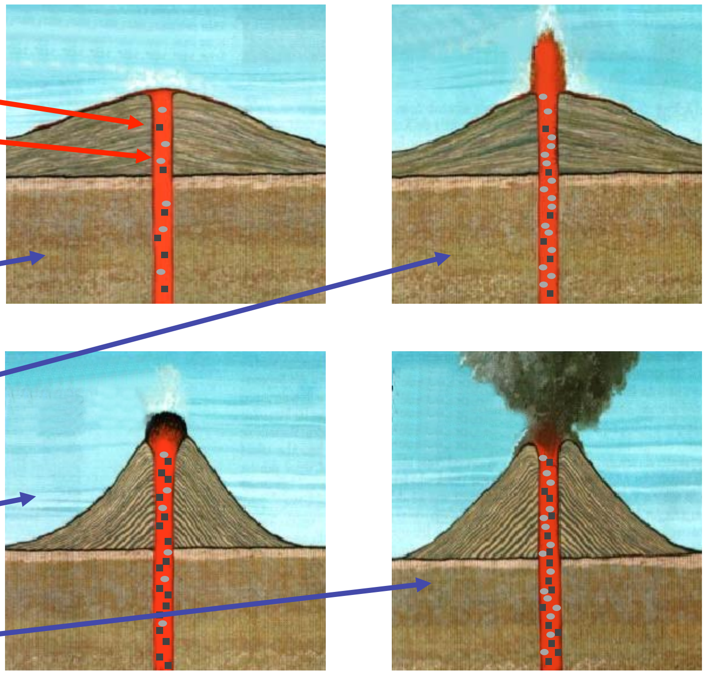

5. Explicate the overall message

summarize in 4-5 sentences
explicate the link to your prior knowledge

Text and picture show 4 types of eruptions in volcanism. Crucial for these types are water and silicon oxide. Wit much water the eruptions are heavy (pictures on the right). With much silicon oxide the lava is viscous and the form of the hill is more steep. Both factors work together.
I had a little prior knowledge because I already knew the text and one of the pictures. In this combination of text and all 4 pictures I now can realize the relations and its easier to imagine the 4 types.

6. What is the relation between text and picture?

The text includes more information. The pictures are missing a legend that shows which icon means water or silicon oxide. Wit a legend the pictures would be also informative for themselves. The pictures are nevertheless necessary, because they additionally show the form of the hill.

In the following you see an example where you can exercise to use the text-picture mapping-strategy. Again, please use the instruction for the strategy on the colored page.

example text-picture combination, e.g. from wikpedia, (text, 1 page long with a meaningful, complementary picture)

What’s next?

Today you learned about three strategies and had a first chance to exercise them. To further strengthen this knowledge we want to work on these strategies with you during the next three weeks.

In this week (from today on) we want you to select two texts that you already come along with in your seminars or lectures or in your everyday life. Work on them by using the 5-steps reading method. To see whether you get along with the strategies or whether you have any difficulties, we ask you to bring those two texts with you to the next seminar session.

In the next week (26.11. – 02.12) you have the opportunity to work on two pictures of your choice from your studies or everyday life. And again we ask you to bring the pictures with you to the following seminar session.

In the third week (03.12. – 09.12.) we will focus on linking text and pictures. Please choose again two examples of text-picture combinations of your study program or everyday life. Please ensure that the picture does not only have decorative function but also includes relevant information. And again we ask you to bring your examples with you to the following seminar session.

The textbook is yours for the next three weeks. At the end of the experiment we nevertheless want to also take a look at this workbook to see whether and how you used the exercises and checklists.

That’s it for today!

Thank you very much for participating and enjoy using the strategies!
