# Supplementary material for: Training for Coherence Formation When Learning From Text and Picture and the Interplay With Learners’ Prior Knowledge
Source: Front Psychol. 2019 Feb 7;10:193. doi: 10.3389/fpsyg.2019.00193 (PMC6374309; doi:10.3389/fpsyg.2019.00193)
Supplement: Supplementary file 2 [file Data_Sheet_2.PDF]

A

# Media in Education
# Meaningful supplement or a waste of time?

an experimental study in the context of the seminar “learning with media”

Dr. Tina Seufert

# Media in Education
# Meaningful supplement or a waste of time?

an experimental study in the context of the seminar “learning with media”

Dr. Tina Seufert

Dear students,

You agreed to participate in our experiment on multimedia learning, thank you very much!

The goal of this workbook is to give you some information on media in education, that should stimulate you to think of pros and cons of media use in education.

In todays seminar lesson we ask you to do the following:

- ➡ Working through the text book
- ➡ Inquiring for additional information in the issue in the internet
- ➡ Discussing the pros and cons in two subgroups to prepare the forums discussion that will follow during the next three weeks.

The single steps will be explained in detail. If you have any questions do not hesitate to ask the investigator.

During the whole experiment we want to ask you to use a code, so that all of your notes and contributions will be recorded anonymously. Please note your Code here:

Code ____________________

Enjoy!

## 1. task: reading

On the following pages you can find articles on “media in education”. There is one basic article on the main forms of media use in education. In addition there are two newspaper articles, in which either sense or nonsense of media use are discussed critically.

Please read the articles carefully and take your time.

## 2. task: web inquiry

While reading the articles you gained first knowledge on the issue “media in education”

The next task is to make a web inquiry for additional information on that issue. You can work with a partner or alone.

The following questions could be helpful:

- In which forms are media used in education?
- Are there any projects in which media use in education is researched?
- What aspects are in favor or in disfavor of using media in education?

The goal is to gather information about the advantages and disadvantages of media use in education and to form your own opinion.
Make notes on important arguments or interesting examples.

<u>20 minutes before the lesson ends you should build 2 groups (A and B). The letter on the front page of this booklet (upper right corner) indicates to which group you belong.</u>

<u>The next section will explain you what to do next….</u>

## 3. task: discuss in pro/con groups

### Task for group A (Pro media use in education):

Today you collected information and arguments on the issue “media in education”. During the next three weeks you are asked to discuss this issue in an online forum.
You now can prepare this online discussion.
Your role as group A is to argue positively for the use of media in education. Together you can now think of all the arguments that you can use during the discussion.

How does the forums discussion work

- [Login information]
- If you click on the link for the forums discussion you can see a window with posts that has already been made by others. You can write new posts, reply on existing ones or upload files. Just make use of all the features (we want to experience the use of media in our own educational setting)
- The forum should be visited regularly during the next three weeks and comments should be added.
- You can also discuss within your group A or B (e.g. who will reply on which statement)

Goal of the forums discussion is to exchange well-grounded arguments and to underline them with your knowledge by using references, articles or texts. Write the arguments in a comprehensive way and in whole sentences and add references to them.

**That’s all for today!**
**Thanks for your participation and enjoy the forum!**

## 3. task: discuss in pro/con groups

## Task for group B (Contra media use in education):

Today you collected information and arguments on the issue "media in education". During the next three weeks you are asked to discuss this issue in an online forum.
You now can prepare this online discussion.
Your role as group B is to argue critically against the use of media in education. Together you can now think of all the arguments that you can use during the discussion.

How does the forums discussion work

- [Login information]
- If you click on the link for the forums discussion you can see a window with posts that has already been made by others. You can write new posts, reply on existing ones or upload files. Just make use of all the features (we want to experience the use of media in our own educational setting)
- The forum should be visited regularly during the next three weeks and comments should be added.
- You can also discuss within your group A or B (e.g. who will reply on which statement)

Goal of the forums discussion is to exchange well-grounded arguments and to underline them with your knowledge by using references, articles or texts. Write the arguments in a comprehensive way and in whole sentences and add references to them.

**That's all for today!**
**Thanks for your participation and enjoy the forum!**
